# Scavenging of copper(II) ions, phosphate(V) ions, and diuron from aqueous media by goethite modified with chitosan or poly(acrylic acid)

**DOI:** 10.1007/s11356-023-27783-8

**Published:** 2023-06-08

**Authors:** Katarzyna Szewczuk-Karpisz, Sylwia Kukowska, Katarzyna Grygorczuk-Płaneta, Bartosz Kondracki, Katarina Jerin, Davor Kovačević

**Affiliations:** 1 Institute of Agrophysics, Polish Academy of Sciences, Doświadczalna 4, 20-290 Lublin, Poland; 2 Chair and Department of Cardiology, Medical University in Lublin, Jaczewskiego 8 (SPSK Nr 4), 20-954 Lublin, Poland; 3 Division of Physical Chemistry, Department of Chemistry, Faculty of Science, University of Zagreb, Horvatovac 102a, 10000 Zagreb, Croatia

**Keywords:** Iron oxide mineral, Coating with polyelectrolytes, Agrochemical components, Adsorption/desorption, Zeta potential study, Aggregation

## Abstract

Goethite was modified by chitosan (CS) or poly(acrylic acid) (PAA) to improve its adsorptive abilities toward components of agrochemicals, i.e., copper ions (Cu), phosphate ions (P), and diuron. The pristine goethite effectively bound Cu (7.68 mg/g, 63.71%) and P (6.31 mg/g, 50.46%) only in their mixed systems. In the one adsorbate solutions, the adsorption levels accounted for 3.82 mg/g (30.57%) for Cu, 3.22 mg/g (25.74%) for P, and 0.15 mg/g (12.15%) for diuron. Goethite modification with CS or PAA did not yield spectacular results in adsorption. The maximum increase in adsorbed amount was noted for Cu ions (8.28%) after PAA modification as well as for P (6.02%) and diuron (24.04%) after CS modification. Both goethite modifications contributed to clear reduction in desorption of pollutants (even by 20.26% for Cu after PAA coating), which was mainly dictated by electrostatic attractive forces and hydrogen bonds formation occurring between macromolecules and impurities. The only exception in this phenomenon was Cu desorption from CS-modified solid—the polymer made it higher (to 95.00%). The Cu adsorption on PAA-modified goethite enhanced solid aggregation and thus facilitated metal cation separation from aqueous media. Consequently, the goethite modification with PAA was considered more promising for environmental remediation.

## Introduction

Expansively growing human population is a major challenge for agriculture. It contributes to the increasing demand for food and thus application of more and more plant protection products and fertilizers improving crop quality and quantity (Kopittke et al. 2019). Excessive use of agrochemicals leads to accumulation of various harmful substances in natural ecosystems. The group of compounds most frequently detected in water-soil environment includes, among others, phosphates (P), copper (Cu), and diuron.

Phosphate fertilizers are applied regularly in agriculture to avoid physiological disorders in plant growth (Xu et al. 2019). This usually leads to eutrophication in neighboring water reservoirs manifested by algal overgrowth and oxygen depletion (Awual 2019a). The P concentration equal to 100 mg/L is high enough to induce this phenomenon (Kumar et al. 2019; Nazari-Sharabian et al. 2018). The phosphates use in various industries has also became larger and larger in recent years. As a result, they are present in industrial sewage and can cause additional environmental pollution due to illegal waste discharges or insufficient treatment (Ganesh et al. 2012). In waters, the mean detected P concentrations are 552.63 ng/L in Nanjing (China), 226.6 ng/L in Shihwa Lake (Korea), and 265.3 ng/L in Sydney (Australia) (Qiao et al. 2022). In the USA, the P level in soils typical of the mid-Atlantic region is 12.5 mg/kg (Maguire et al. 2005), whereas soils from poultry production areas contains even 568 mg/kg of these compounds (Hooda et al. 2001). Cu is classified as a micronutrient essential for proper plant growth. However, at higher concentrations this is a toxic element having negative effect on organisms (Ju et al. 2019). Anthropogenic activities including application of copper-based fertilizers, mining, and metal ore processing contribute to excessive accumulation Cu in the environment (Rehman et al. 2019). The Cu toxicity occurs when its concentration exceeds 50 mg/L in sandy soils or 150 mg/L in silty-clay or clay soils (Droz et al. 2021). The maximum Cu amounts detected in surface waters were 9.0-261.0 μg/L, while in groundwater, 64.0-2,783.0 μg/L (ATSDR 2022). Diuron (3-(3,4-dichlorophenyl)-1,1-dimethylurea) is applied to control unwanted weeds. Due to its high persistence in the environment, it is considered a serious threat for soils and waters (Egea et al. 2017). Diuron is metabolized by soil bacteria and fungi to several toxic metabolites, among which DCA (3,4-dichloroaniline) has the highest nephrotoxicity and hepatotoxicity (Mohammed et al. 2020). UE has set the maximum permissible residue level of diuron in the soil to 0.01 mg/kg for fruits, vegetables, cereals, and sugar crops; 0.02 mg/kg for nuts and oil crops; and 0.05 mg/kg for herbs, tea, coffee, herbal infusions, spices, and animal products (Szewczuk-Karpisz et al. 2021a). So far, its average concentration detected in Australia was 397 μg/kg in (Stork et al. 2008), in turn in the USA, 380 μg/kg (Field et al. 2003).

The harmful effects of phosphates, copper ions, and diuron can be reduced by their adsorption on the solid surfaces. As a consequence, their leaching into neighboring waters and bioavailability can be reduced significantly. Nowadays, many researchers are looking for environmentally friendly, biodegradable, and low-cost adsorbents that are able to bind large amounts of pollutants and can be applied for environmental remediation. For Cu detection and removal from wastewater, several composite materials based on silica were developed and described. There are mesoporous silica with 5-tert-butyl-2-hydroxybenzaldehyde thiosemicarbazone (THTB) ligand (Awual 2015), mesoporous silica with Schiff base ligand containing nano-composite adsorbent (NCA) (Awual et al. 2015b), mesoporous silica monoliths with (3-(3-(methoxycarbonyl)benzylidene) hydrazinyl)benzoic acid ligand (Awual et al. 2013), mesoporous silica monolith with 3-(((5-ethoxybenzenethiol)imino)methyl)-salicylic acid (EBMS) ligand (Awual et al. 2015c), mesoporous silica monolith with N,N-bis(salicylidene)1,2-bis(2-aminophenylthio)ethane ligand (Awual et al. 2014a), mesoporous silica with sulfur donor containing organic ligand of ammonium (4-chlro-2-mercaptophenyl)carbamodithioate (ACMPC) (Awual et al. 2016), mesoporous silica with N,N-disalicylidene-4,5-dimethyl-phenylenedene (DDPD) ligand (Awual 2017), reusable ligand anchoring stable composite (Awual 2019b), porous silica with 2-methyl-8-quinolinol ligand (Awual et al. 2019), mesoporous silica with 4-tert-Octyl-4-((phenyl)diazenyl)phenol (TPDP) ligand (Salman et al. 2023a), and porous silica with 4-dodecyl-6-((4-(hexyloxy)phenyl)diazenyl)benzene-1,3-diol (DPDB) ligand (Kubra et al. 2021). Composite material for simultaneous removal of Cu and lead(II) ions, that is, 6-((2-(2-hydroxy-1-naphthoyl)hydrazono)methyl)benzoic acid (HMBA) embedded onto mesoporous silica monoliths, was also investigated (Awual et al. 2014b). Similar materials were successively applied for thulium(III) (Kubra et al. 2023), lutetium(III) (Hasan et al. 2023a, 2023b), palladium(II) (Awual and Yaita 2013), and cadmium(II) (Hasan et al. 2023a, 2023b) capturing. Silica-based composites with immobilized organic ligands are very promising as well as show high sensitivity and sorption capacity toward metal ions.

The second group of composites used to remove metals and other pollutants are those prepared on the basis of soil minerals and macromolecular compounds. So far, the effect of macromolecular compound on adsorption capacity of minerals or mineral composites toward toxic substances has been tested several times. Szewczuk-Karpisz et al. (2021b) examined impact of poly(acrylic acid) on Cu adsorption on the carbon-mineral composites with metallic elements. The same team determined the impact of bacterial exopolysaccharide on accumulation of Cu, chromium (Cr), and carboxin on montmorillonite (Szewczuk-Karpisz et al. 2022). Fijałkowska et al. (2021) described adsorption mechanism of lead (Pb) on kaolinite and montmorillonite modified with cationic and anionic polyacrylamide (PAM). Medykowska et al. (2022) examined the removal of diclofenac and heavy metal ions (Pb, zinc (Zn)) using zeolitic materials in the systems containing also PAA 2000 or PAA 240 000. The impact of CS coating on mineral adsorptive abilities has rarely been investigated. Most researchers applied chitosan as a substrate to synthesize new materials (Da Silva Alves et al. 2021). Sutirman et al. (2018) performed the chemical and physical modification of CS in order to improve removal of metal ions from aqueous solutions. Chen et al. (2020) obtained chitosan-carboxylmethyl starch composites and examined their adsorption capactity relative to Cu. Gu et al. (2019) prepared chitosan-lignosulfonate composite for removal of dyes and metals from wastewater. Salman et al. (2023b) investigated CS-coated cotton fiber composite for dye removal. Many researchers used CS for hydrogel synthesis, e.g., magnetic chitosan/poly(vinyl alcohol) beads (Zhu et al. 2012), methacrylamide/chitosan crylogels (Kundakci 2020), and polyacrylamide-g-chitosan gel (Da Silva et al. 2020). Goethite coated with PAA or CS has not been reported yet.

Goethite (α-FeOOH) is one of the most stable iron oxyhydroxides in nature, present in almost all soil types (Liu et al. 2013), as well as a waste product of hydrometallurgical processes, e.g., zinc production. In the second case, goethite may be an environmental problem and limit light penetration and photosynthesis when discharged into waters (Szewczuk-Karpisz and Wiśniewska 2014). Goethite application for adsorption purposes creates an opportunity to manage it and reduce potential threat to ecosystems (Szewczuk-Karpisz et al. 2019). Since the specific surface area of this mineral varies in a large range and may be as low as 10 m^2^/g (Liu et al. 2013), it seems necessary to modify it and improve its sorption capacity.

Therefore, in this work, an attempt was made to coat goethite with two polymeric substances of different properties and origins, i.e., CS and PAA, to explore new potential soil conditioners or adsorbents for contaminated surface or groundwater treatment. The adsorption capacity of the goethite with and without macromolecular compounds was examined toward Cu, P, and diuron, in the systems containing one or two of them at the same time. The mechanism of Cu/P/diuron adsorption on goethite, coated or uncoated with CS or PAA, was explored based on the study including isotherms and kinetics, adsorption data modeling, potentiometric titration, and Fourier transform infrared spectroscopy (FTIR). The strength of pollutants binding was estimated during the desorption study. Zeta potential and turbidimetric measurements were used to determine the stability of the goethite suspension with and without adsorbed polymers/pollutants. It was predicted that goethite modification with CS or PAA will improve its adsorption abilities toward various substances due to introduction of additional functional groups together with polymers. The presence of the polymer may also stimulate the aggregation of goethite with adsorbed impurities, which is desirable during the purification of aqueous systems. It is worth emphasizing that the presented composites were prepared using only safe, environmentally friendly substrates which made them suitable for environmental remediation. CS is obtained by chemical or enzymatic deacetylation of chitin (the main component of the cuticle of crustaceans) (Saheed et al. 2021), whereas PAA is a non-toxic polymer produced by the radical polymerization of acrylic acid (Szewczuk-Karpisz et al. 2021b).

The presented issue can be considered highly innovative and important from the point of view of water and soil protection. Simultaneous adsorption of copper ions, phosphate ions, and diuron on the goethite has not been described in the literature. The impact of the goethite modification of PAA or CS on its adsorption capacity toward selected impurities and aggregation also remains unknown. This paper is part of the search for new means to remediate degraded ecosystems. It also describes how waste goethite is managed in order to enable closed-loop operation.

## Experimental

### Materials

Goethite (CAS 20344-49-4) was applied as an adsorbent in the experiments. Chitosan (CAS 9012-76-4) of medium molecular weight and poly(acrylic acid) (CAS 9003-01-4) were used as macromolecular compounds for goethite coating. CS average molecular weight (M_w_), determined by size exclusion chromatography (SEC-MALS), was equal to 411 kDa (Matusiak et al. 2022). Conductometric titration with HCl indicated that the deacetylation degree (DD) of the applied CS was 75%. The average molecular weight of PAA was 2000 Da. Both polymers were delivered by Sigma Aldrich and were not further cleaned. Copper ions, phosphate ions, and diuron (C_9_H_10_Cl_2_N_2_O, CAS 330-54-1) were used as pollutants. Copper(II) chloride dihydrate, CuCl_2_ × 2H_2_O (CAS 10125-13-0) was applied as a source of copper ions and monosodium phosphate, (CAS 7558-80-7) as a source of phosphate ions. The formulas and structures of adsorbent and adsorbates are summarized in Table 1. Before the experiments, the goethite was washed out using demineralised water to a conductivity of < 2 μS/cm.

**Table 1:**
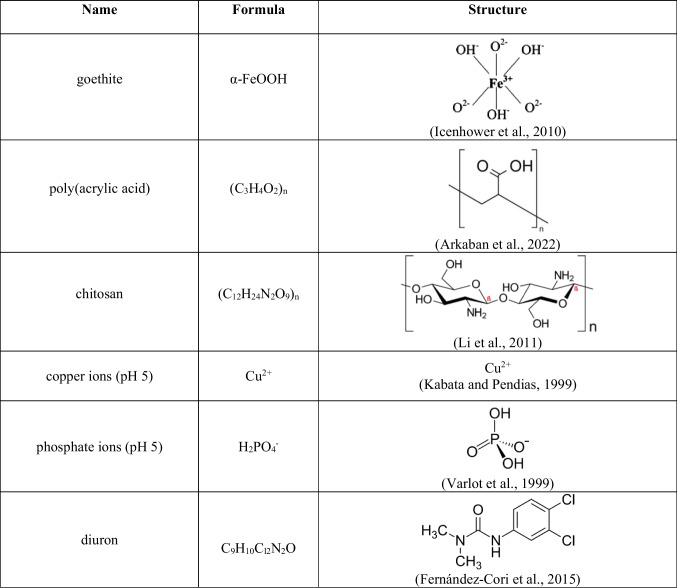
Characteristics of adsorbent and adsorbates

To adjust pH value of the examined systems, hydrochloric acid (HCl, CAS 7647-01-0) and sodium hydroxide (NaOH, CAS 1310-73-2) were used. In the determination of polymer concentration sulfuric acid (H_2_SO_4_, 98%, CAS 7664-93-9) and hyamine 1622 (0.004 mol/dm^3^, CAS 121-54-0) were applied. Sodium chloride (NaCl, CAS 7647-14-5) was used as a supporting electrolyte. All the chemicals were delivered by Sigma Aldrich.

The concentrations of stock solutions of copper ions and phosphate ions as well as CS and PAA were 1000 mg/L. In turn, the concentration of the stock solution of diuron, due to its limited solubility in water, was 10 mg/L.

## Methods

### Goethite characterization

The goethite sample was characterized using Fourier transform infrared spectroscopy (FTIR), scanning electron microscopy (SEM-EDS), nitrogen adsorption/desorption, energy dispersive X-ray analyses (EDX), and potentiometric titration.

Determination of goethite surface groups was performed using a Nicolet 6700 Fourier-transform infrared (FTIR) spectrometer (Thermo Scientific) equipped with a Smart Orbit diamond-attenuated reflectance attachment. The spectra were recorded in the range of 4000–400 cm^-1^, at 4 cm^-1^ intervals, from 128 scans, and were corrected with a linear baseline using the OMNIC v.8.2 software (Thermo Scientific).

The morphology of the goethite was observed using a scanning electron microscopy (SEM) (Phenom ProX, PiK Instruments) equipped with an energy dispersive spectrometer (EDS). The applied acceleration voltage was 10 kV. The analyses were carried out in a low vacuum mode without coating the sample with conductive layer.

Based on nitrogen adsorption/desorption isotherms, measured using an analyzer 3Flex (Micromeritics, USA), the specific surface area (S_BET_) and porosity parameters (total pore volume—*V*_p_, pore diameter—*D*) of goethite were determined. Specific surface area was calculated using the Brunauer-Emmet-Teller (BET) equation as well as the capacity of monolayer formed on the mineral surface. The porosity parameters were estimated using nitrogen desorption isotherm. Before the measurement, the sample was dried and out-gassed (105 °C, 12 h).

Energy dispersive X-ray analyses (EDX-7200, Shimadzu) were used to determine elemental composition of goethite.

Potentiometric titration was applied to estimate the point of zero charge (pH_pzc_) of the mineral. The titration was performed using an automatic burette (Titrino 702 SM, Metrohm) and 0.1 M sodium hydroxide (NaOH) as the titrant, at pH values of 3-10. The surface charge density (*σ*_*0*_) was calculated using the method developed by Janusz (1994).

### Goethite modification

Goethite modification was performed by its coating with polymers via adsorption. At the beginning, the samples (10 mL) containing 0.04 g of the goethite, the supporting electrolyte (0.01 mol/L NaCl) and the polymer (50 or 100 mg/L of CS/PAA) were prepared. After the pH adjustment to the value of 5, the adsorption was conducted for 24 h. This time ensured the achievement of equilibrium state in the examined systems. After process completion, the samples were centrifuged to separate modified goethite from the solution. The amounts of adsorbed polymers were determined based on the difference in their concentration in the solution before and after the adsorption, using the following formula (Ościk 1969):

1 $$\Gamma =\frac{C_{ads}\bullet V}{m}$$

where *C*_*ads*_—the adsorbed amount of the polymer (*C*_*ads*_ *= C*_*0*_ *− C*_*eq*_) [mg/L], *V*—the system volume [L], *m—*the weight of the goethite [g].

In turn, the efficiency, *E*, of the polymer adsorption was calculated using the equation (Szewczuk-Karpisz et al. 2022):

2 $$E=\frac{C_A}{C_0}\bullet 100\%$$

where C_A_—the concentration of polymer adsorbed on the goethite [mg/L], C_0_—the initial polymer concentration in the sample [mg/L].

The concentration of CS was determined using the method developed by Albalasmeh et al. (2013), in turn the concentration of PAA—by the method with hyamine 1622 (Kang et al. 2013). During the adsorption on solid surfaces, macromolecular compounds behave differently than ions and small molecules. They assume specific conformations including structures like “loops” and “tails” and one polymer chain can interact with several active sites (Fijałkowska et al. 2019). Therefore, the adsorbed amounts of CS/PAA on goethite are presented only as a histogram.

### Study on pollutant adsorption in the single systems

To examine kinetics of the pollutant adsorption on the goethite, a series of samples was prepared. They consisted of 0.04 g of the mineral and 10 mL of the solution containing 0.01 mol/L NaCl as a supporting electrolyte and the selected pollutant (copper ions/phosphate ions/diuron). The applied concentration of copper ions/phosphate ions was 100 mg/L, while that of diuron, 5 mg/L. After the pH adjustment to the value of 5, the adsorption was started and conducted for 5, 15, 30, 60, 120, 180, 300 min using a rotator (Multi RS-60, Biosan, 30 rpm). When the process was complete, the solid with adsorbed impurities was separated from the solution by centrifugation (3000 rpm, 10 min, centrifuge SBS-LZ-4000/20-6, Steinberg Systems) and using syringe filters (0.22 μm, AlfaTec Technology). The concentration of phosphate in the obtained clear solutions was determined by ion chromatography (ICS-1100, Dionex), the concentration of copper ions—by an atomic absorption spectrometer working in the technique with a graphite cuvette (contrAA 900, Analytic Jena), whereas the concentration of diuron—by high pressure liquid chromatography (Dionex Ultimate 3000 equipped with a diode array detector).

To prepare adsorption isotherms, the samples were prepared as mentioned above. The applied concentration of ions was in the range of 10-200 mg/L, whereas that of diuron was in the range of 1-9 mg/L. After the samples preparation, their pH value was adjusted to 5 using 0.1 mol/L HCl or 0.1 mol/L NaOH and a pH meter (CX-505, Elmetron), and the adsorption was started. The process was conducted under conditions of continuous mixing (rotator Multi RS-60, Biosan, 30 rpm), for 24 h. This time was chosen based on the kinetics study results (it ensured equilibrium state in the examined systems). The concentration of pollutants in the obtained clear solutions was analyzed in the same way as during the study on adsorption kinetics.

As in the case of polymer adsorption, the adsorbed amount of copper ions/phosphate ions/diuron on the goethite was calculated based on the difference in their concentrations in the solution before and after the adsorption process using Eq. (1). The efficiency of pollutant adsorption was determined using Eq. (2).

### Study on the pollutant adsorption in the mixed systems

During the adsorption study in the mixed systems, the samples were prepared using 0.04 g of the goethite as well as 10 mL of the solution containing 0.01 mol/L NaCl as a supporting electrolyte and 50 mg/L of two different substances, either two pollutants or one pollutant and one polymer. In this way, it was possible to measure the mutual influence of impurities on their adsorption on the goethite surface, as well as the influence of CS/PAA on the adsorption of impurities. The adsorption process and the determination of copper ions/phosphate ions/diuron concentration were performed in the same way as described in the section above.

### Study on pollutant desorption

The goethite with adsorbed impurities, obtained in the adsorption study with or without polymers, was dispersed in an aqueous solution at pH 5 (10 mL, the pH value of desorbing solution was adjusted using 0.1 mol/L HCl). Then the desorption process was conducted for 1 h under continuous shaking (rotator Multi RS-60, Biosan, 30 rpm). After that, the solid was separated from the solution using centrifuge (3000 rpm, 10 min, centrifuge SBS-LZ-4000/20-6, Steinberg Systems) and syringe filter (0.22 μm, AlfaTec Technology), and the concentration of pollutants was analyzed in the same way as during “adsorption study.” The desorption degrees (%) of copper ions/phosphate ions/diuron from pristine and modified goethite were calculated using the equation:

3 $$\%\textrm{des}=\frac{C_{\textrm{des}}}{C_A}\bullet 100\%$$

where *C*_des_ is the desorbed concentration of the ion or herbicide [mg/L].

### Adsorption data modeling

Experimental adsorption isotherms were modeled using Langmuir, Freundlich and Redlich-Peterson models. The kinetics data was fitted to the pseudo I-order (PFO) and pseudo II-order (PSO) equations. The Langmuir (Eq. 4) and Freundlich (Eq. 5) models are described as (Foo and Hameed 2010):

4 $${q}_e=\frac{q_m{K}_L{C}_e}{1+{K}_L{C}_0}$$

5 $${q}_e={K}_F{C}_e^{1/n}$$

where *K*_F_ [mg/g(mg/L)^-1/nF^]—the Freundlich parameter, *K*_L_ [L/mg]—the Langmuir parameter, *q*_e_ [mg/g]—the equilibrium adsorption capacity, *C*_e_ [mg/L]—the equilibrium liquid phase concentration; *q*_m_ [mg/g]—the maximum adsorption capacity in Langmuir model, *n*—the Freundlich constant related to adsorption intensity.

The Redlich-Peterson isotherm model (Eq. 6) is defined as (Kumara et al. 2014):

6 $${q}_e=\frac{K_{RP}\bullet {C}_e}{1+{a}_{RP}^{\beta }}$$

where *K*_RP_—the Redlich–Peterson adsorption capacity constant [L/mg], *a*_RP_—the Redlich–Peterson isotherm constant [(L/mg)^β^], *β*—the exponent.

In turn, the pseudo I-order (Eq. 7) and pseudo II-order (Eq. 8) equations are expressed as (Ho and McKay 1998, 2002):

7 $$\frac{d{q}_t}{dt}={k}_1\left({q}_e-{q}_t\right)$$

8 $$\frac{d{q}_t}{dt}={k}_2{\left({q}_e-{q}_t\right)}^2$$

where *q*_*e*_—the adsorbed amount at equilibrium [mg/g]; *q*_*t*_—the adsorbed amount after time *t* [mg/g]; and *k*_*1*_ [1/min] and *k*_*2*_ [g/mg·min]—the rate constants.

### Zeta potential study

The zeta potential values of the goethite with and without polymers/pollutants were calculated based on electrophoretic mobility values measured using ZetaPlus (Brookhaven Instruments Corporation). At first, 0.001 g of goethite was added to 100 mL of sodium chloride solution (0.01 mol/L). The pH of the solution was adjusted to the value of 5 by adding hydrochloric acid. After 10 min of sonication, the suspension was divided into several parts, to which a certain volume of impurities and polymers was added to obtain their final concentration of 50 mg/L for copper ions/phosphate ions/polymers and 5 mg/L for diuron. Such prepared suspensions were stirred on the magnetic stirrer (HI 190M, Hanna Instruments) for 30 min to allow adsorption to occur. In the systems with electrostatic repulsion between adsorbate and adsorbent, this time was extended to 20 h to ensure that equilibrium was established. If the pH of the suspensions changed after adding polymer or pollutant, it was restored using several drops of 0.1 mol/L HCl or 0.1 mol/L NaOH. The pH of the systems was measured using a pH-meter (827 pH lab, Metrohm). After the suspension stirring for the selected time, the electrophoretic mobility of the goethite was measured.

### Stability study

The stability of the goethite suspensions, with and without pollutants/polymers, was measured using a UV-Vis spectrophotometer (Jasco V-530) at a wavelength of 500 nm. The samples were prepared by adding 0.01 g of the goethite the solution of supporting electrolyte (0.01 mol/L NaCl). After 10 min of sonication, the selected substance (polymer, pollutant, or polymer + pollutant) was added and the pH value of the samples was adjusted to 5. The concentration of copper ions/phosphate ions and CS/PAA in the examined systems was 50 mg/L, whereas that of diuron, 5 mg/L. One stability measurement lasts 1 h, during which the absorbance was measured every 10 s.

### Statistical analysis

All the adsorption/desorption measurements were made in triplicate, in turn zeta potential measurements were repeated 10 times. The measurement uncertainty was determined by calculation of standard deviation (Statistica software and Microsoft Office Excel). The measurement error did not exceed 5%.

## Results and discussion

### Goethite characteristics

Goethite is an iron oxyhydroxide of the formula α-FeOOH, mainly composed of iron hydroxide (80-90%) and water (10-20%). In its crystals, oxygen and hydroxyl anions are packed hexagonally in arrays, whereas all iron atoms are coordinated octahedrally (Table 1) (The Editors of Encyclopaedia Britannica 1998). The EDX analyses indicated that the applied goethite was composed of the following: iron (Fe)—about 32% and oxygen (O)—about 67%, as well as trace amounts of sulfur (S), zinc (Zn), copper (Cu), manganese (Mn), and strontium (Sr).

**Fig. 1:**
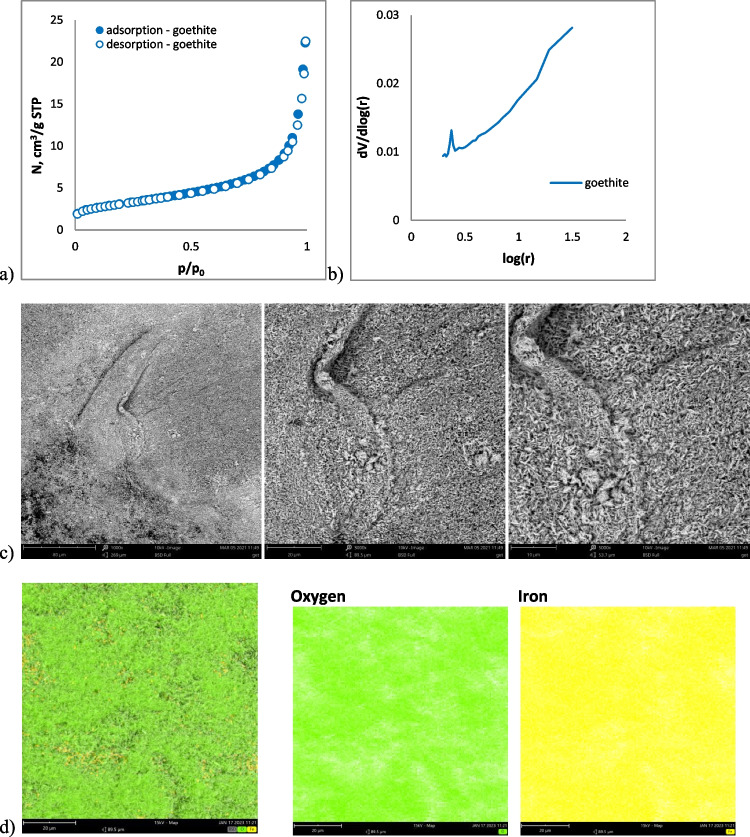
Goethite characteristics: nitrogen adsorption/desorption isotherms (**a**), pore size distribution (**b**), SEM image (**c**) with elemental composition maps (**d**)

Textural parameters of the goethite were rather poor. The N_2_ adsorption/desorption experiment indicated that its *S*_*BET*_ parameter equaled 11 m^2^/g, and the *V*_p_ was 0.034 cm^3^/g (Table 2). The obtained nitrogen adsorption/desorption isotherms represented type IV with the H3 hysteresis loop (Fig. 1a). Such an isotherm type describes a multilayer adsorption with capillary condensation on mesoporous materials. The H3-type hysteresis loop is present in aggregate, parallel-corrugated pellets with slit-like pores. The calculated pore size distribution (Fig. 1a) indicated mesoporous character of the goethite. The *D*_PSD_ value was 4.7 nm, whereas *D*_4V/A_ was 12.2 nm. The obtained *S*_*BET*_ parameter corresponded with that of synthetic goethite prepared by Ulatowska (2022).

**Table 2:** Characteristics of the goethite (*S*_*BET*_, the specific surface area; *V*_*p*_, the total pore volume; *D*_*PSD*_, the pore diameter at the maximum of PSD obtained from the N_2_ desorption branch and *D*_*4V*/A_, the pore diameter calculated using 4V/A; *pH*_pzc_, the point of zero charge determined by potentiometric titration)

Sample	*S*_*BET*_ [m^2^/g]	*V*_*p*_ [cm^3^/g]	*D*_PSD_ [nm]	*D*_4V/A_ [nm]	pHpcz
Goethite	11	0.034	4.7	12.2	8.2

The SEM images showed that the goethite particles were needle-like and elongated (Fig. 1b).

Potentiometric titration results showed that the point of zero charge (pH_pzc_) of the goethite was equal to 8.2 (Szewczuk-Karpisz et al. 2019). This means that at pH = 8.2, the surface charge of the goethite is equal to 0 and then neutral groups, ≡Fe_2_OH, are dominant on its surface. At pH < 8.2, the mineral is positively charged because ≡FeOH^1/2+^ and ≡Fe_3_OH^1/2+^ groups prevail. At pH > 8.2, the goethite surface charge is negative due to the domination of ≡FeO^1/2-^ and ≡Fe_3_O^1/2-^ moieties on the surface. The above specific groups are created on the goethite surface because, according to Pauling’s rules, oxygens are singly, doubly, and triply coordinated and all iron atoms are octahedrally coordinated in its structure (Tadanier and Eick 2002).

### Adsorption mechanism of copper ions/phosphate ions on the pristine goethite

At pH 5, at which adsorption study was performed, copper ions occurred as Cu^2+^, whereas phosphate ions were mainly in the form of H_2_PO_4_^-^ (Kabata-Pendias and Pendias 1999; Varlot et al. 1999). The goethite surface was positively charged (Szewczuk-Karpisz et al. 2019).

**Fig. 2:**
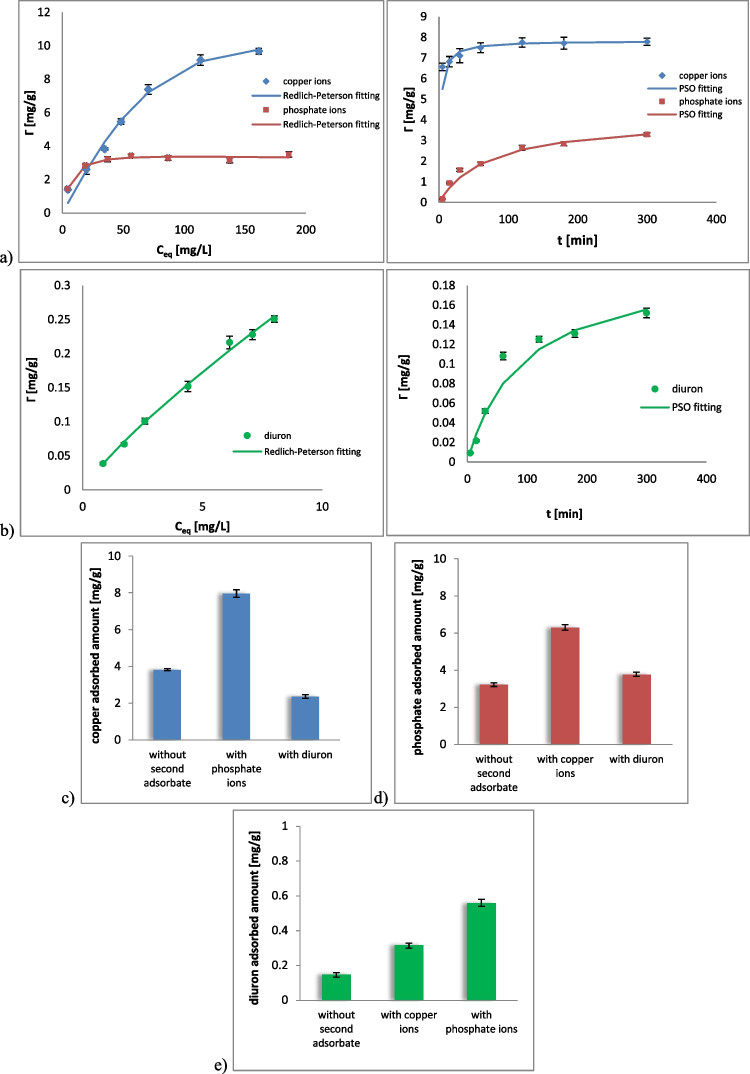
Adsorption isotherms and kinetics of copper ions, phosphate ions (**a**) and diuron (**b**) on goethite with the fitting to Redlich-Peterson and pseudo second-order (PSO) models as well as comparison of adsorbed amounts of copper ions (**c**), phosphate ions (**d**), and diuron (**e**) in the systems containing one or two pollutants simultaneously

In Fig. 2a, the time effect on the Cu and P adsorption on goethite is presented. At the beginning, there was a rapid increase in adsorbed amount associated with the ions bonding on external surface of mineral crystals. Then, after 100 min for Cu and 300 min for P, the kinetic curves increased more slowly due to the adsorbate diffusion into internal sites. Experimental kinetic data were best fitted to the pseudo II-order equation (*R*^2^ was in the range of 0.959–0.999, Table 3). This means that Cu and P adsorption on goethite could be described as chemisorption. Cu ions can form inner- and outer-sphere complexes on the goethite surface. In the first case, the metal ions interact with the surface directly based on covalent or ionic bonds (specific adsorption), whereas in the second one, at least one water molecule is located between the system components and the adsorption process is of coulombic character (non-specific adsorption) (Perelomov et al. 2013; Grossi and Sparks 1994). Outer-sphere surface complexation prevails at low pH, while, the inner-sphere one, at high pH values (Liu et al. 2013). Phosphate ions adsorption on the goethite is mainly based on the surface complexation (Fe-O-P-O-Fe type) and formation of hydrogen bridges. The replacement of hydroxyl groups by adsorbing anions also occurs in the system (Liu et al. 2013). Due to the fact that goethite is positively charged at pH 5, there is electrostatic attraction between the solid particles and the H_2_PO_4_^-^ ions (Ler and Stanforth 2003). Surface complexation reactions occurring between goethite active sites and ions can be described as follows (Jaiswal et al. 2013; Katz 2017):

9 $$\equiv {SOH}^0+{H}^{+}\leftrightarrow \equiv {SOH}_2^{+}$$

10 $$\equiv {SO H}^0\leftrightarrow \equiv {SO}^{-}+{H}^{+}$$

11 $$\equiv {SO}^0+{Me}^{2+}\leftrightarrow \equiv {SO Me}^{+}+{H}^{+}$$

12 $$\equiv {SO}^0+{H}^{+}+{OxAn}^{2-}\leftrightarrow \equiv S{(OxAn)}^{1-}+{H}_2O$$

13 $$\equiv {SO H}^0+{Cat}^{2+}\leftrightarrow \equiv {SO}^{-}\_{Cat}^{2+}+2{H}^{+}$$

14 $$\equiv {SOH}^0+{H}^{+}+{An}^{-}\leftrightarrow \equiv {SOH}^{2+}\_{An}^{-}$$

**Table 3:** Isotherm and kinetics parameters of pollutants’ adsorption on pristine goethite

		Langmuir			Freundlich			Redlich-Peterson			
		*q*_m_ [mg/g]	*K*_L_ [L/mg]	*R*^2^	*n*	*K*_F_ [mg/g (mg/L)^-1/nF^]	*R*^2^	*K*_RP_ [L/mg]	*a*_RP_ [(L/mg)^β^]	*β*	*R*^2^
Isotherm parameters	Copper ions	13.983	0.014	0.911	1.713	0.122	0.972	0.159	0.00045	1.148	0.997
	phosphate ions	3.476	0.220	0.994	0.969	0.077	0.775	0.508	0.10231	1.069	0.999
	Diuron	0.849	0.052	0.893	1.177	0.456	0.998	0.092	1.07043	0.271	0.999
		Pseudo I-order				Pseudo II-order					
		*q*_*e*_ [mg/g]	*k*_1_ [1/min]		*R*^2^	*q*_e_ [mg/g]		*k*_2_ [g/mg·min]		*R*^2^	
Kinetics parameters	Copper ions	1.072	0.044		0.821	7.834		0.060		0.999	
	Phosphate ions	8.568	0.076		0.810	4.089		0.003		0.959	
	Diuron	7.578	0.027		0.909	0.203		0.053		0.955	

where *Me*^*2+*^—the metal ions involved in inner-sphere reaction, *Cat*^*2+*^—the metal ions involved in outer-sphere reaction, *OxAn*^*2-*^—the anions involved in inner-sphere reaction, *An*^*-*^—the anions involved in outer-sphere reaction.

The experimental isotherms of Cu and P adsorption, presented in Fig. 2b, were best fitted to the Redlich-Peterson model (*R*^2^ was in the range of 0.997–0.999, Table 3). The β parameter for both ions was close to unity, which meant that their adsorption was localized (ions did not move on the surface and one ion interacted with one active site) (Majd et al. 2022). Ulatowska (2022) reported that arsenic(V) adsorption on synthetic goethite was best described by Dubinin-Radushkevich and pseudo second-order models. Jaiswal et al. (2013) indicated that Cu and Cd adsorption on synthetic goethite was best fitted to Freundlich and pseudo second-order equations. Similarly, cobalt (Co) and nickel (Ni) adsorption on natural iron oxide and synthetic goethite was best described by Freundlich and pseudo second-order models (Nangah et al. 2019).

**Fig. 3:**
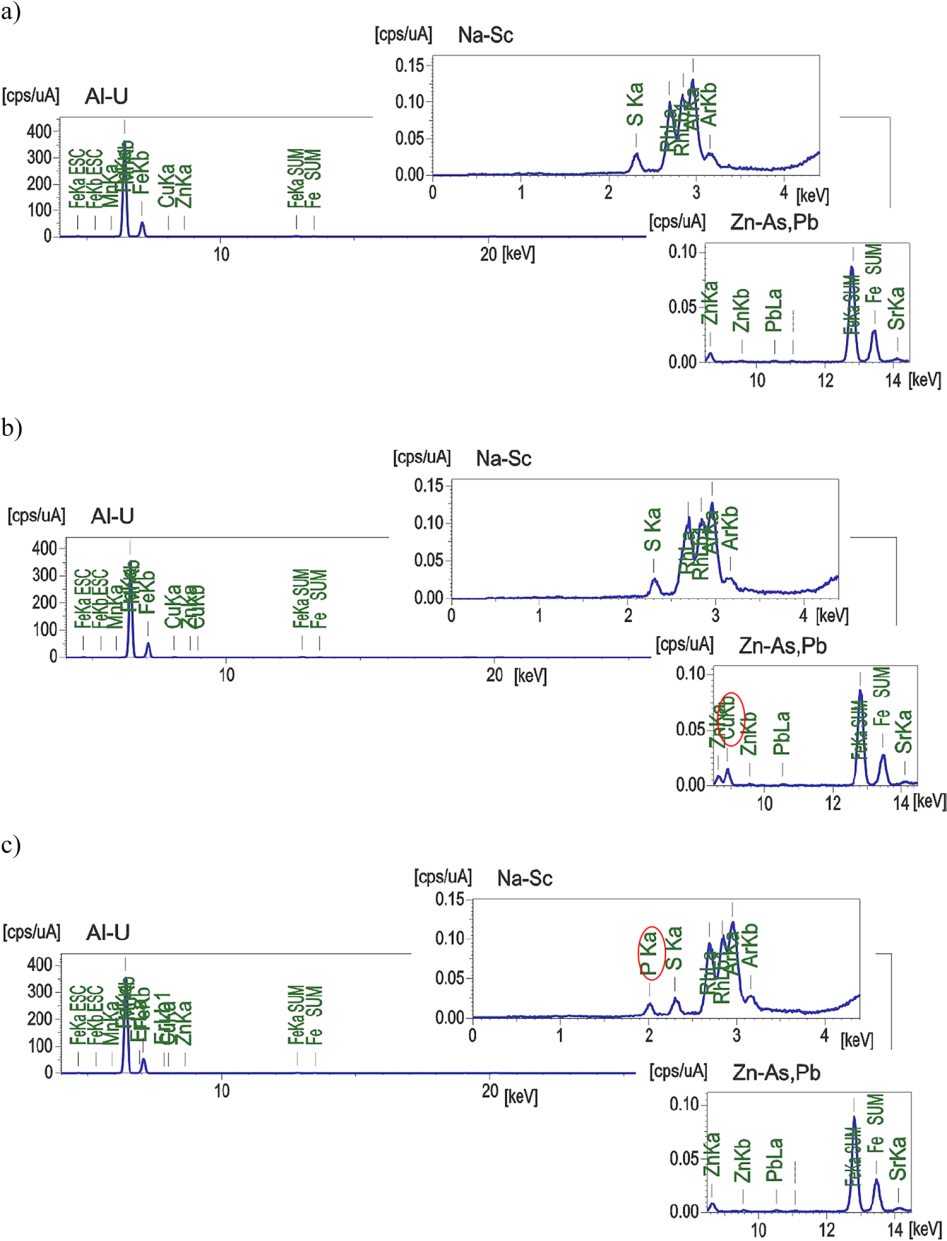
EDX results of goethite without pollutants (**a**) as well as goethite with adsorbed copper ions (**b**) or phosphate ions (**c**)

The EDX analyses (Fig. 3) confirmed that copper and phosphate ions were adsorbed on the goethite surface. There are additional peaks in the spectra registered for goethite after ions adsorption, not visible in the spectrum for the goethite before the process. There are peak around 9 keV corresponding to Cu (Fig. 3b) and peak around 2 keV corresponding to P (Fig. 3c).

**Fig. 4:**
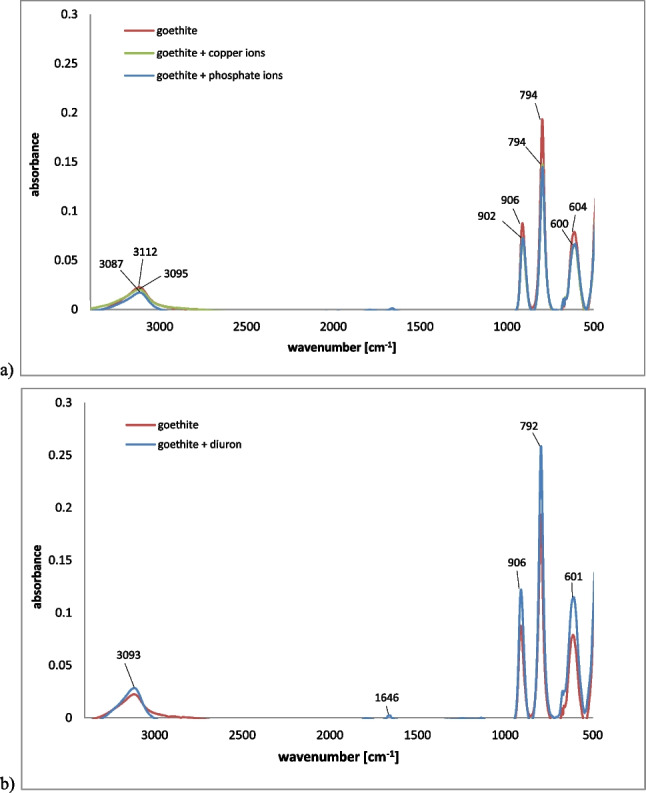
FTIR spectra of goethite with and without adsorbed copper/phosphate ions (**a**) as well as goethite with and without adsorbed diuron (**b**)

After ions adsorption, the changes occurred also in the FTIR spectrum of goethite (Fig. 4a). The spectrum of pristine goethite consisted of the following bands at: 3112 cm^-1^ (the typical OH stretching band of oxyhydroxides), 906 cm^-1^ and 794 cm^-1^ (which can be attributed to the FeOOH bending), 604 cm^-1^ (the Fe–O stretching). After the Cu and P adsorption, the 3112 cm^-1^ bands shifted to 3095 cm^-1^ and 3087 cm^-1^, respectively. Moreover, the intensity of the bands at 904, 794, and 604 cm^-1^ was decreased. All these changes were probably dictated by the interactions of copper and phosphate ions with Fe–O–H and Fe–O groups (Salimi et al. 2018).

It is also worth mentioning that the adsorption capacity of the goethite was slightly higher toward Cu than toward P. For initial ions concentration of 50 mg/L, the adsorbed amount was 3.82 mg/g and 3.22 mg/g for copper and phosphate ions, respectively (Fig. 2). The higher adsorption capacity can be explained by different size of ions. Ionic radius of Cu^2+^ is 0.73 Å, whereas that of H_2_PO_4_^-^, 2.13 Å (Kadim and Gamaj 2020). Smaller ions can be bounded in larger amount on unit surface area of the adsorbent.

The measured adsorbed amounts of Cu and P on the pristine goethite were considered rather low. For the initial ions concentration of 200 mg/L, the obtained adsorbed amounts were 9.78 mg/g (19.36%) and 3.50 mg/g (7.00%), respectively. A comparative study indicated that a few other mineral adsorbents had similar, also unsatisfactory, adsorptive abilities. What is more, the adsorption capacity of synthetic goethite was much higher than that of natural one, applied in this study (Table 4). This means that the modification of goethite improving its adsorptive abilities is highly needed.

**Table 4:** Comparison of adsorption capacity toward copper ions, phosphate ions, and diuron of various solids (*C*_*0*_, the initial concentration of the pollutant; *m*_*a*_, the adsorbent dose)

Pollutant	Adsorbent	Conditions	Adsorption capacity (mg/g)	References
Copper ions	Goethite	C_0_ = 200 mg/L pH = 5	9.78	This study
	Synthetic goethite	C_0_ = 5-25 mg/L pH = 5	357.14	Jaiswal et al. 2013
	Kaolinite	C_0_ = 50 mg/L pH = 5.7	4.40	Bhattacharyya and Gupta 2006
	Bentonite	C_0_ = 200 mg/L pH = 5	5.30	Olu-Owolabi et al. 2010
	Zeolite	C_0_ = 10-200 mg/L pH = 5	5.91	Álvarez-Ayuso et al. 2003
	Montmorillonite	C_0_ = 1000 mg/L pH = 6.5	1.58	Akpomie and Dawodu 2015
	Red claystone	C_0_ = 10-220 mg/L pH = 5.5	20.3	Musso et al. 2014
Phosphate ions	Goethite	C_0_ = 200 mg/L pH = 5	3.50	This study
	Kaolinite	C_0_ = 250 mg/L pH = 5.45	0.32	Moharami et al., 2013
	Bentonite	C_0_ = 250 mg/L pH = 5.45	0.28	Moharami and Jalali 2013
	Zeolite (natural)	C_0_ = 200 mg/L pH= 7	2.15	Sakadevan and Bavor 1998
	Natural iron oxide coated sand	C_0_ = 5-30 mg/L pH = 5	0.88	Boujelben et al. 2008
	Hydrous niobium oxide (Nb_2_O_5_·4H_2_O)	m_a_ = 2 g/L pH = 5	5.10	Rodrigues and da Silva 2009
	Magnetic diatomite clay	C_0_ = 25 mg/L pH = 8	9.39	Chen et al. 2016
Diuron	Goethite	C_0_ = 5 mg/L pH = 5	0.25	This study
	Kaolinite	C_0_ = 5 mg/L pH = 4.8	0	Polati et al. 2006
	Na-montmorillonite	C_0_ = 5 mg/L pH = 9.9	0.034	Polati et al. 2006
	Fly ash	C_0_ = 5 mg/L pH = 7	0.146	Quirantes et al. 2017

### Adsorption mechanism of diuron on the pristine goethite

The pK_a_ value of diuron is 13.55, so at pH 5, it has a slight positive charge (Wong et al. 2016). Under these conditions, the amount of the positive species (DH^+^) is about 10% (Deng et al. 2012).

Figure 2b presents the kinetics of diuron adsorption on the goethite surface. A rapid increase in pesticide adsorbed amount was observed up to 120 min. Then, the kinetics curve was close to the equilibrium. Experimental data were best fitted to the pseudo II-order equation with *R*^2^ equal to 0.955 (Table 3), which indicated chemical character of the tested process. The adsorption of organic molecules on goethite involves mainly electrostatic effects, ligand exchange, and hydrogen bonding (Liu et al. 2013). The donor-acceptor interactions between hydroxyl groups of goethite and amino/carbonyl moieties of diuron are dominant (Szewczuk-Karpisz et al. 2021a). The experimental isotherms were best described by Redlich-Petersen model (*R*^2^ = 0.999, Table 3). The β parameter was close to 0, which meant that the adsorption of diuron met the assumptions of Freundlich model (the pesticide molecules show some mobility in the adsorption layer, and they occupied centers of the highest adsorption energy at first).

The adsorption of diuron was visible in the FTIR spectrum of goethite (Fig. 4b). The band at 3112 cm^-1^ was shifted to 3093 cm^-1^, whereas the bands at 904 cm^-1^, 794 cm^-1^, and 604 cm^-1^ were intensified. All these changes were associated with the interactions of above mentioned groups of diuron and goethite. The band at 1646 cm^-1^, that appeared in the spectrum, was attributed to the bending vibration of –OH or N=C groups present in the pesticide structure (Correa-Navarro et al. 2020).

Minerals are usually characterized by low adsorption capacity toward pesticides due to high hydrophobicity of adsorbate (Hundal et al. 2001). The largest adsorbed amount of diuron, noted for its initial concentration of 9 mg/L, was 0.25 mg/g (11.15%). Kaolinite, montmorillonite, and fly ash bound fewer molecules of diuron (Table 4). The solid modification with polymeric compounds should enhance its interactions with hydrophobic substances.

### Adsorption of copper ions/phosphate ions/diuron on the goethite in the mixed systems

In the systems containing two types of adsorbates (two ions or ion + pesticide), adsorption capacity of pristine goethite was different than that in the systems with only one adsorbate (Fig. 2). The addition of 50 mg/L of phosphate ions to 50 mg/L of copper ones increased the Cu adsorbed amount from 3.82 mg/g (30.57%) to 7.96 mg/g (63.71%). At pH 5, a certain amount of HPO_4_^2-^ ions was present in the system. They could form specific bridges between positive goethite surface and copper cations and, as a result, weaken electrostatic repulsion between positively charged system compounds. Enhanced adsorption of Cu, Zn, Cd, and Pb on goethite was also observed in the presence of sulfate (Swelund et al. 2009). The addition of diuron had different effect on heavy metal adsorption, that is, it limited the tested process. After the addition of 50 mg/L of diuron to 50 mg/L of copper ions, the Cu adsorbed amount changed from 3.82 mg/g (30.57%) to 2.36 mg/g (18.86%). This was probably dictated by the competition between positive diuron molecules and copper cations for goethite active sites.

Regarding the phosphate ions adsorption, the addition of 50 mg/L of Cu to 50 mg/L of phosphate ions strengthened the binding of the latter. The phosphate adsorption increased from 3.22 mg/g (25.74%) to 6.31 mg/g (50.46%). The HPO_4_^2-^ ions, a small amount of which was present in the system together with H_2_PO_4_^-^, were probably responsible for the formation of the second, third, and next layers of pollutants on goethite. One HPO_4_^2-^ ion interacted with one adsorbed Cu ion and another Cu ion from the solution, which contributed to larger metal adsorption. The addition of 50 mg/L of diuron to 50 mg/L of phosphate ions also increased the phosphate binding from 3.22 mg/g (25.74%) to 3.78 mg/g (30.21%). Between these pollutants hydrogen bonds were created, which contributed to adsorption of additional P amounts.

The addition of ions, copper, or phosphate (50 mg/L), to 5 mg/L of diuron increased the adsorbed amount of this pesticide from 0.15 mg/g (12.15%) to 0.318 mg/g (25.47%) and 0.558 mg/g (44.65%), respectively. Bivalent cations can form cation-herbicide or cation-herbicide-cation complexes, which can precipitate and thus contribute to higher adsorption of herbicide on the solid surface. Metal ions interact with two chlorine atoms attached to an aromatic ring of diuron, i.e., the site of high electronegativity capable of attracting cations (Das Chagas et al. 2019). On the other hand, phosphate ions are attracted to protonated region of diuron molecules by electrostatic forces. In this case, HPO_4_^2-^ species could also act as specific bridges between positive goethite particles and positive diuron molecules, which increased the herbicide adsorption.

### Modification of the goethite with polymeric substances

**Fig. 5:**
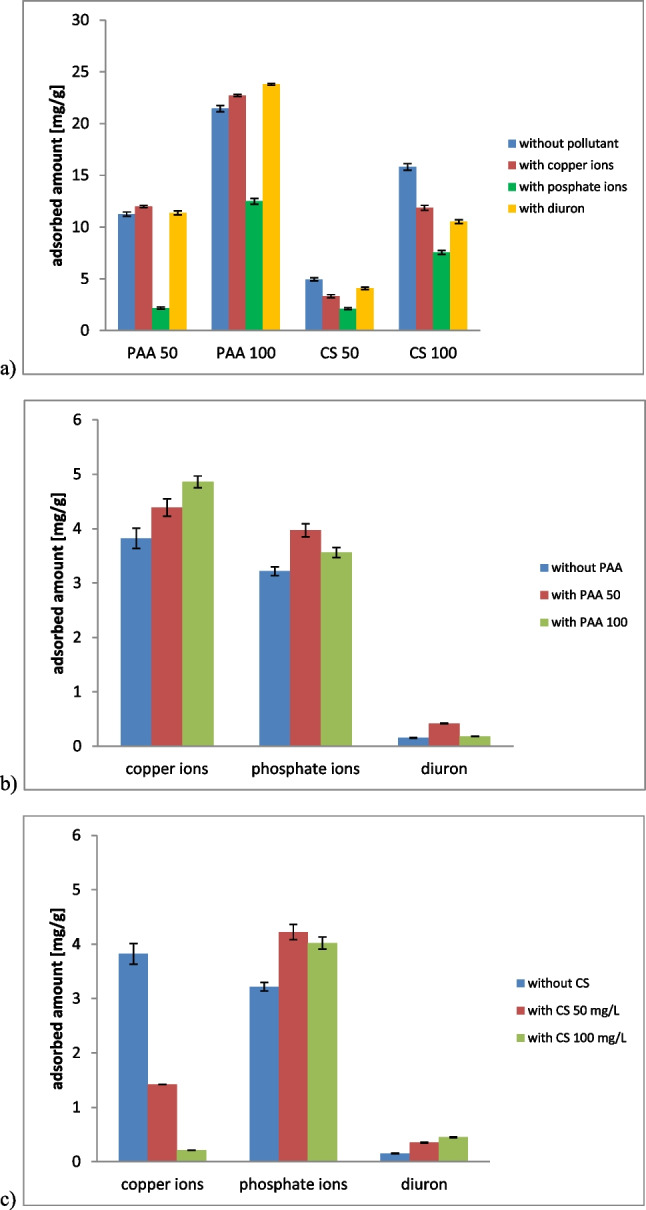
Adsorbed amounts of poly(acrylic acid) and chitosan on goethite with and without pollutants (**a**) as well as adsorbed amounts of copper ions, phosphate ions, and diuron on goethite modified with PAA (**b**) or CS (**c**) with the concentration of 50 or 100 mg/L

Goethite modification with macromolecular compounds was performed based on the adsorption process. Figure 5a shows the adsorbed amounts of PAA and CS on the mineral for the polymer initial concentrations of 50 or 100 mg/L. Both polyelectrolytes were adsorbed on the goethite surface efficiently. Larger adsorbed amounts were noted for PAA due to attractive electrostatic conditions. They were equal to 11.25 mg/g (90.00%) and 21.46 mg/g (85.83%) for 50 mg/L and 100 mg/L initial concentrations, respectively. On the other hand, the CS-adsorbed amounts were 4.95 mg/g (39.63%) and 15.82 mg/g (63.27%) for the same initial concentrations. The PAA adsorption on goethite was based on electrostatic attraction and hydrogen bonding between positively charged solid particles and negative PAA macromolecules. In turn, for chitosan attachment to the mineral, the interactions between goethite hydroxyl groups and CS amino groups were responsible (Munagapati et al. 2017). Between positive CS chains and positive solid particles there was also electrostatic repulsion, which hindered their mutual contact.

**Fig. 6:**
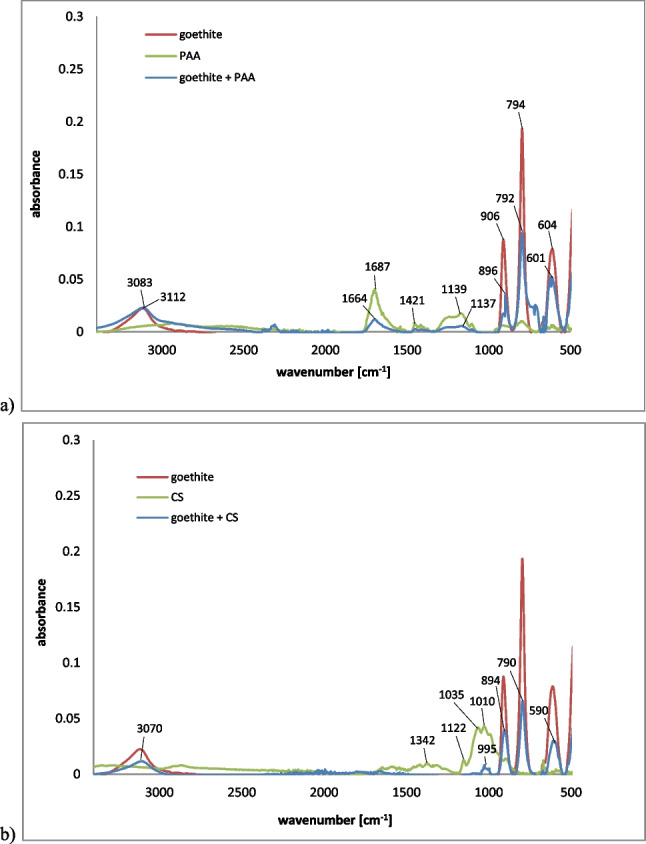
FTIR spectra of goethite, poly(acrylic acid), and goethite with adsorbed poly(acrylic acid) (**a**) as well as goethite, chitosan and goethite with adsorbed chitosan (**b**)

The FTIR spectra of the goethite with and without PAA and CS as well as of the applied polymers are presented in Fig. 6. The bands in the PAA spectrum (Fig. 6a) are as follows: 1687 cm^-1^ (can be assigned to C=O stretching), 1421 cm^-1^ (C-O stretching coupled with O-H in-plane bending), 1139 cm^-1^ (the C-O stretching). Due to the PAA adsorption, in the spectrum of goethite + PAA, the additional bands around 1664 cm^-1^ and 1137 cm^-1^ were visible. Moreover, the bands near 906 cm^-1^, 794 cm^-1^, and 604 cm^-1^ were significantly decreased and shifted to 896 cm^-1^, 792 cm^-1^, and 601 cm^-1^, respectively. This was caused by the poly(acrylic acid) interaction with Fe–O–H and Fe–O groups of the goethite (Dong et al. 1998; Najim et al. 2012).

The spectrum of CS (Fig. 6b) consisted of the following bands: 2830 cm^-1^ (attributed to –NH and –OH groups stretching), 1342 cm^-1^ (CH_3_ deformations), 1122 cm^-1^ (asymmetric stretching of C-O-C), 1035 cm^-1^, and 1010 cm^-1^ (C-O stretching). The spectrum of the goethite modified with chitosan showed significant decreased bands at 906 cm^-1^, 794 cm^-1^, and 604 cm^-1^ (compared to the spectrum of the pristine goethite). Moreover, they were shifted to the regions of: 894 cm^-1^, 790 cm^-1^, and 590 cm^-1^, respectively (Queiroz et al. 2015; Lustriane et al. 2018). This was the evidence of the interactions occurring between the goethite hydroxyl groups and chitosan amino moieties (Munagapati et al. 2017).

Based on these results, it can be stated that, due to the goethite modification with chitosan, additional groups (amino and hydroxyl ones) were introduced to the solid surface. In turn, the mineral modification with poly(acrylic acid) contributed to both change in surface charge sign of the goethite and introduction of carboxylic groups.

**Fig. 7:**
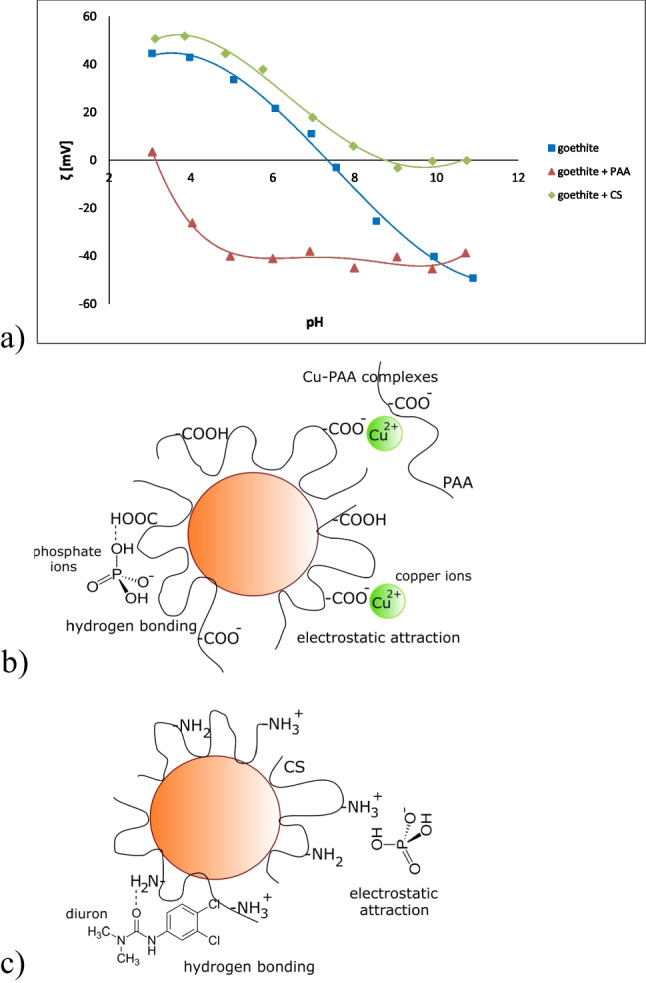
Zeta potential of goethite suspension with and without PAA or CS (**a**) as well as scheme of interactions occurring in the system containing PAA-modified goethite (**b**) and CS-modified goethite (**c**) together with impurities

To confirm goethite coating with the selected polymers, the zeta potential values of the mineral with and without PAA/CS were determined (Fig. 7). In the system without polymeric substances, the goethite isoelectric point (pH_iep_) was about 7.4. In the PAA presence (50 mg/L), electrokinetic potential was negative in almost entire pH range, and the pH_iep_ value was shifted toward the value of 3.1. On the other hand, after the chitosan addition (50 mg/L), zeta potential of the mineral became positive over entire tested pH range, and the pH_iep_ was close to 9. Such significant changes in the slipping plane charge, observed in the presence of PAA or CS, was the evidence for goethite coating with selected polymers.

### Modification of the goethite with polymeric substances in the presence of pollutants

The pollutants presence influenced the adsorptive modification of the goethite (Fig. 5a). Copper ions and diuron (with the concentration of 50 mg/L) slightly increased the adsorbed amount of PAA on goethite. For initial polymer concentration of 50 mg/L, it increased from 11.25 mg/g (90.00%) to 12.00 mg/g (96.00%) and 11.37 mg/g (91.00%) with Cu and diuron, respectively. On the other hand, for initial PAA concentration of 100 mg/L, its adsorption changed from 21.46 mg/g (85.83%) to 22.71 mg/g (90.83%) and 23.79 mg/g (95.17%) with Cu and diuron, respectively. Positively charged ions and molecules were involved in the formation of second polymer adsorption layer. They simultaneously interacted with polymer chains adsorbed and those from the solution and create intermolecular complexes. PAA can also form intramolecular complexes with divalent ions (when one ion interacts with two carboxylic groups of one polymer chain). These complexes had more coiled structure than PAA molecules and, as a result, their larger amount can be adsorbed on the goethite surface (Fijałkowska et al. 2019). The phosphate ions presence affected PAA adsorption in the opposite way. A clear decrease in the adsorption rate from 11.25 mg/g (90.00%) to 2.17 mg/g (17.3%) (for initial PAA concentration of 50 mg/L) and from 21.46 mg/g (85.83%) to 12.50 mg/g (50.00%) (for initial PAA concentration of 100 mg/L) was observed. This was dictated by the competition between negatively charged phosphate ions and negatively charged polyelectrolyte molecules for active sites on the goethite surface.

All applied pollutants reduced the chitosan adsorption on goethite. In the Cu presence (50 mg/L), the CS-adsorbed amount decreased from 4.95 mg/g (39.64%) to 3.32 mg/g (26.55%) (when initial concentration was 50 mg/L) as well as from 15.82 mg/g (63.27%) to 11.86 mg/g (47.45%) (when initial concentration was 100 mg/L). Similarly with phosphate ions, a decrease to 2.11 mg/g (26.54%) and 7.55 mg/g (30.18%) was noted for the initial CS concentrations of 50 and 100 mg/L, respectively. Diuron reduced CS-adsorbed amount to the values of 4.09 mg/g (32.73%) and 10.52 mg/g (42.09%) when initial polymer concentration was 50 mg/L and 100 mg/L, respectively. All molecules and ions of pollutants, definitely smaller in size than the polymer macromolecules, reached the surface as first and block some active sites of the adsorbent.

### Adsorption mechanism of copper ions/phosphate ions/diuron on the modified goethite

The goethite modification with PAA affected the adsorption of all pollutants (Fig. 5b). There was a slight increase in the Cu adsorbed amount from 3.82 (30.57%) to 4.39 mg/g (35.14%) and 4.86 mg/g (38.85%), after PAA addition of 50 mg/g and 100 mg/L, respectively. A certain amount of Cu cations is attracted electrostatically by the PAA macromolecules due to their negative charge. The Cu-PAA complexes formed in the solution, could also be adsorbed on the solid surface. As a consequence, the additional amount of metal ions was accumulated in the adsorption layer (Szewczuk-Karpisz et al. 2021a). In the case of phosphate ions, the increase in their adsorbed amount was also clear after PAA coating. Then, their adsorbed amount changed from 3.22 mg/g (25.74%) to 3.97 mg/g (31.76%) and 3.56 mg/g (29.48%) for 50 and 100 mg/L PAA. Probably, under these conditions, ions interacted with PAA by hydrogen bonding, which contributed to higher adsorption of phosphate. In the contrast, in the presence of 100 mg/L of PAA, the phosphate adsorbed amount decreased to the value of 3.09 mg/g (24.79%). Such a high concentration of the polymer reflected in a very strong electrostatic repulsion between PAA and phosphate ions. As a result, they repelled each other and the ions adsorption was reduced. For diuron adsorption, the impact of the goethite modification with PAA was positive, but significant increase was noted only for lower PAA concentration. The diuron adsorption changed from 0.15 mg/g (12.15%) to 0.42 mg/g (33.77%) and to 0.18 mg/g (14.58%) for 50 mg/L and 100 mg/L of PAA, respectively. This was associated with electrostatic attraction between positively charged diuron molecules and dissociated carboxylic groups of the polymer.

The goethite modification with chitosan, regardless of the applied concentration of the polymer, caused an dramatic decrease in the Cu adsorption (Fig. 5c). The Cu adsorbed amount was reduced from 3.82 mg/g (30.57%) to 1.42 mg/g (11.43%) and 0.21 mg/g (1.71%) for the CS concentrations of 50 mg/L and 100 mg/L, respectively. This phenomenon was caused by strong competition between positively charged chitosan and metal ions. The pK_a_ parameter of chitosan is in the range of 6.39-6.51 (Wang et al. 2006), which means that at pH 5 most amino groups within chitosan chains are protonated. On the goethite covered with CS, P, and diuron were better adsorbed. The phosphate adsorption increased from 3.22 mg/g (25.74%) to 4.22 mg/g (33.72%) (for 50 mg/L of CS) and 4.02 mg/g (32.16%) (for 100 mg/L of CS). The adsorbed amount of herbicide changed from 0.15 mg/g (12.15%) to 0.35 mg/g (27.73%) and 0.45 mg/g (36.19%) for 50 mg/L and 100 mg/L of CS, respectively. Phosphate ions interacted with protonated amino groups (-NH_3_^+^) by electrostatic attractive forces (Kim et al. 2022). On the other hand, diuron could form hydrogen bonds with hydroxyl groups of CS as well as amino groups (-NH_2_), small amounts of which were also present in chitosan macromolecules at pH 5 (Pavinatto et al. 2005).

### Binding strength of copper ions/phosphate ions/diuron on the pristine and modified goethite

Desorption degrees of the pollutants measured in the examined systems are summarized in Table 5. The results obtained for pristine goethite indicated that Cu was desorbed in the highest amount from the solid surface (26.14%). This was associated with acidic character of the applied desorbing solution. Acids, such as sulfuric acid, nitric acid, and hydrochloric acid, are very efficient desorbing agents (Abdo Allozy and Khairil Juhanni 2020). They usually cause protonation of solid surface and enhanced electrostatic repulsion between adsorbent and adsorbate (Isaac and Siddiqui 2022). Desorbed amounts of phosphate ions and diuron were significantly lower (8.01% and 9.89%, respectively). During phosphate desorption, the hydroxyl groups could replace adsorbed anions (Zhang et al. 2015). In the mixed systems, i.e., containing two types of pollutants, the desorption degrees were different. In most solutions, they were lower than those observed in the systems with one impurity. Copper ions were desorbed in the amount of 21.57% and 21.21% in the presence of phosphate ions and diuron, respectively. Diuron desorption was also smaller with other pollutants—1.76% with Cu and 5.70% with phosphate ions. In the case of phosphate ions, the presence of metal ions and herbicide increased their desorption to the values of 18.39% and 9.43%, respectively. This meant that the binding of phosphate ions to the surface was weaker under these conditions.

**Table 5:** Desorption degree [%] of copper ions, phosphate ions, and diuron from the single systems and mixed systems containing another pollutant or macromolecular compound

Desorbed pollutant	Single systems	Mixed systems						
		With copper ions	With phosphate ions	With diuron	With PAA 50	With PAA 100	With CS 50	With CS 100
Copper ions	26.14	-	21.57	21.21	12.19	5.88	95.00	91.67
Phosphate ions	8.01	18.39	-	9.43	5.61	6.62	7.46	4.84
Diuron	9.89	1.76	5.70	-	1.48	3.91	4.52	0.76

In the systems containing modified goethite, desorption degrees were generally lower than those measured for pristine goethite, with the exception of copper ions. The CS polymer layer enhanced desorption of Cu (91.67–95%), but reduced that of phosphate anions (4.84–7.46%) and diuron (0.76–4.52%). The PAA modification decreased desorption of all tested impurities. Then, desorption degrees for copper ions were in the range of 5.88–12.19%, those of phosphate ions, 5.61–6.62%, whereas those of diuron, 1.41–3.91%. Reducing desorption of pollutants is highly desirable during the remediation of polluted ecosystems.

### Zeta potential values of the goethite with pollutant or pollutant/polymer

Adsorption of pollutants affected goethite zeta potential significantly (Table 6). Cu contributed to increase in absolute values of positive zeta potential, diuron decreased absolute values of positive zeta potential, whereas adsorption of phosphate ions led to the negative zeta potential values. In the case of Cu, the non-specifically adsorbed ions, located in the outer Helmholtz layer were responsible for increase of absolute zeta potential values (Birdi 2003). In the system containing diuron, the reduction in absolute values of positive electrokinetic potential was associated with the presence of herbicide fragments containing chlorides (of high electronegativity) in the slipping plane area. The negative zeta potential of goethite observed after the addition of phosphate ions was mainly dictated by the adsorption of HPO_4_^2-^. Then, one negative charge interacted with the solid surface, whereas the second one was located in the slipping plane area.

**Table 6:** Zeta potential values of goethite suspension with and without PAA, CS, and pollutants in one or two adsorbate systems at pH 5

System	*ζ* [mV]	Standard deviation
Pristine goethite	20.78	2.03
Goethite + copper ions	22.43	1.07
Goethite + phosphate ions	−26.93	1.64
Goethite + diuron	0.16	1.6
Goethite + PAA	−40.08	1.39
Goethite + CS	44.5	1.73
Goethite + PAA + copper ions	−21.23	0.77
Goethite + PAA + phosphate ions	−36.44	1.01
Goethite + PAA + diuron	−33.7	1.07
Goethite + CS + copper ions	43.87	0.8
Goethite + CS + phosphate ions	39.65	0.32
Goethite + CS + diuron	39.25	0.62

In the presence of both polymer and pollutant, zeta potential of goethite was mainly determined by the adsorbed macromolecular compound. In the systems with PAA, zeta potential was negative, whereas in those with CS, positive. The polymer chains formed structures like “loops” and “tails” on the goethite surface, which contained dissociated carboxylic groups (in the case of PAA) or protonated amino groups (CS). Exactly these moieties were located in the slipping plane area and affected its charge. The increase in absolute values of negative zeta potential was also dictated with the offset of slipping plane as a result of the adsorption of poly(acrylic acid) (Fijałkowska et al. 2019; Wiśniewska et al. 2015).

### Stability mechanism of the goethite suspension with and without pollutants/polymers

**Fig. 8:**
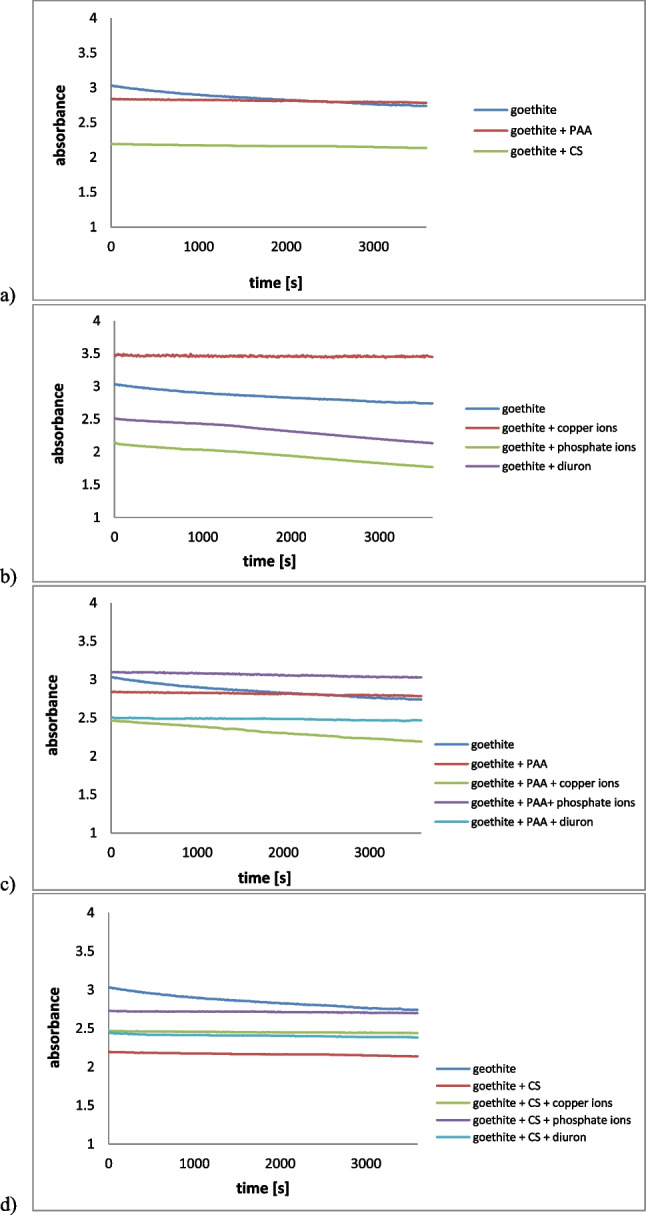
Stability of goethite suspension with and without PAA/CS (**a**) PAA/pollutants (**b**), and CS/pollutants (**c**)

At pH 5, the aqueous suspension of pristine goethite had relatively high stability—the absorbance remained constant during the measurement (Fig. 8). Positively charged particles were surrounded by chloride ions coming from the supporting electrolyte, which limited their mutual contact (electrostatic stabilization) (Szewczuk-Karpisz et al. 2015).

Most of the goethite suspensions containing polymers and/or pollutants were also relatively stable. A clear reduction in the system stability, which is highly desirable during wastewater treatment, was noted only for the suspensions containing goethite/phosphate ions or PAA-modified goethite/copper ions. Then, the absorbance gradually decreased over time. Phosphate anions, added to the system, neutralized positive charge of the positive particles and, as a consequence, the electrostatic repulsive forces were weakened. On the other hand, copper ions acted as bridges between adsorbed PAA macromolecules, which facilitated the goethite aggregation.

## Conclusions

Goethite was modified in order to improve its adsorption capacity toward pollutants as well as its subsequent use as an adsorbent in environmental remediation. In this way, an attempt was made to manage waste mineral.

Due to low specific surface area, pristine goethite was an effective adsorbent of Cu and phosphate ions only in the systems containing both pollutants at the same time. Goethite modification with macromolecular compounds introduced additional functional groups to the solid and affected its surface charge. The PAA coating slightly increased Cu adsorption due to the formation of Cu-PAA complexes. It also enhanced the binding of phosphates based on the hydrogen bonds creation. The CS modification made the adsorption of phosphates and diuron higher. P ions interacted with protonated amino groups (-NH_3_^+^) of CS by electrostatic attractive forces, whereas diuron could form hydrogen bonds with amino (-NH_2_) and hydroxyl groups of this polymer. In the systems with modified goethite, the pollutant desorption was generally lower, which meant that polymers contributed to strong immobilization of impurities. The only exception was the CS effect on Cu desorption—its intensification was observed. The presence of macromolecular compound did not destabilize the system due to relatively high absolute values of zeta potential of the mineral. A clear aggregation was noted only in the system containing pristine goethite and phosphates, which was caused by the neutralization of positive solid charge by anions, and in the system containing Cu and PAA-modified goethite, where divalent ions created “bridges” between adsorbed polymer chains. Taking all these results into account, the goethite modification with PAA was considered more promising for environmental remediation.
